# The Effect of Particle Shapes on the Field-Dependent Rheological Properties of Magnetorheological Greases

**DOI:** 10.3390/ijms20071525

**Published:** 2019-03-27

**Authors:** Norzilawati Mohamad, Ubaidillah, Saiful Amri Mazlan, Seung-bok Choi, Siti Aishah Abdul Aziz, Masataka Sugimoto

**Affiliations:** 1 Engineering Materials and Structures (eMast) iKohza, Malaysia-Japan International Institute of Technology (MJIIT), Universiti Teknologi Malaysia, Jalan Sultan Yahya Petra, 54100 Kuala Lumpur, Malaysia; mnorzilawati@gmail.com (N.M.); aishah118@gmail.com (S.A.A.A.); 2 Mechanical Engineering Department, Faculty of Engineering, Universitas Sebelas Maret, Jl. Ir. Sutami 36A, Kentingan, Surakarta, 57126 Central Java, Surakarta, Indonesia; 3 National Center for Sustainable Transportation Technology, 40132 Bandung, Indonesia; 4 Department of Mechanical Engineering, Smart Structures and Systems Laboratory, Inha University, Incheon 402-751, Korea; 5 Graduate School of Organic Materials Science, Yamagata University, 4-3-16, Jonan, Yonezawa 992-8510, Japan; sugimoto@yz.yamagata-u.ac.jp

**Keywords:** magnetorheological grease (MRG), bidisperse MRG, particle shape, spherical particle, plate-like particle, rheological properties, transient response

## Abstract

The transient response of magnetorheological (MR) materials, in general, is very important for design consideration in MR-based devices. Better response to magnetic fields is beneficial for a better response rate to the electrical current applied in the electromagnetic coil. As a result, MR-based devices would have a high response to external stimuli. In this work, the principal characteristics of magnetorheological greases (MRGs) which have two different particle shapes are experimentally investigated. One type of particle distributed in the grease medium is conventional spherical-shaped carbonyl iron (CI) particles, while the other is plate-like CI particles made using a high-energy rotary ball mill from spherical CI particles. A set of bidisperse MRG samples are firstly prepared by adjusting the weight percentage of the plate-like CI particles and mixing with the spherical CI particles. Subsequently, three important properties of MRGs in terms of their practical application are measured and compared between the two different particle shapes. The field-dependent apparent viscoelastic properties of the prepared MRG samples are measured, followed by the field-dependent storage and loss moduli using an oscillatory shear rheometer. In addition, the transient response time, which indicates the speed in the actuating period of MRGs, is measured by changing the strain amplitude. Then, a comparative assessment on the three properties are undertaken between two different particle shapes by presenting the corresponding results in the same plot. It is shown that the bidisperse MRG with plate-like CI particles exhibits an increase in the initial apparent viscosity as well as stiffness property compared to the MRG with spherical particles only.

## 1. Introduction

In recent years, many works have been undertaken on magnetorheological (MR) fluids. MR fluids have attracted the interests of many researchers owing to their prompt ability to form chain structures reversibly from liquid-like to solid-like within milliseconds by applying an external magnetic field [1,2,3,4,5]. The study on the rheological properties, such as shear stress, apparent viscosity, storage modulus, and MR effect, have been extensively investigated [6,7,8,9,10,11,12]. Recently, interest on the transient response of MR fluids has arisen to understand the mechanism of chain structure formation and to develop fast dynamic systems in real applications [13,14]. MR fluids possesses excellent performance in terms of field-dependent rheological changes and are hence applied in several engineering fields, including automotive shock absorbers [15]. However, due to severe sedimentation and leakage problems, MR fluid is limited in its effective usage in many other practical applications [16,17,18,19]. Therefore, MRG has been introduced to overcome the drawbacks of MR fluids. One of the salient properties of MRG is to have a higher MR effect over MR fluids and MR elastomers [20,21]. Recently, in order to use MRG in practical applications, systematic investigations on prototype devices utilizing MRG have been actively conducted. Similar to MR fluids, most prototype applications are oriented towards semi-active control applications, such as seismic dampers, machine dampers, vehicle dampers, and clutches [22,23]. Several works on MRG have validated that the settling problem of the iron particles can be eliminated by utilizing grease which has thixotropic properties as a medium [24,25,26]. These properties restrict any reversible flow in the application devices without the presence of external force [27,28]. In addition, some other advantages of MRG include self-sealing, thick viscosity, anti-wear properties, low friction, and robustness to extreme temperatures and pressures [29,30]. However, most research works on MRGs so far have focussed on the field-dependent rheological properties at various magnetic field intensities [24]. It has been demonstrated from these works that MRGs can produce very high yield stress without any sedimentation behaviour. In addition, it has been shown that the field-dependent rheological properties of MRGs can be enhanced by adding certain additives, such as nanosized carbon [31]. Besides works on the field-dependent properties, the controllability of the initial viscosity of MRGs by addition of small amounts (5 wt%) of kerosene has been examined [32]. It has also been shown that the yield stress of MRGs is dependent solely on the magnetic particles [26]. For example, the MR effect can be increased by up to 950% by utilizing 70 wt% of magnetic particles. 

On the other hand, a few works on the field-dependent rheological properties of MRGs which have different particle shapes have been undertaken. Recently, a comparative work on the yield stress between two different particle shapes was investigated [33]. It has been found that the optimal weight percentage of the plate-like and spherical magnetic particles to provide the highest yield stress were 30 and 70 wt%, respectively. It has been identified that the utilization of plate-like particles requires a lower concentration to achieve the same level of yield stress as with spherical particles due to the long direction and anisotropy axes of the plate-like particles [34]. In other words, the plate-like magnetic particles are easily magnetized and have a smaller demagnetization factor as the external magnetic field is applied. This phenomenon leads to the formation of strong and stable chain structures compared to the spherical magnetic particles [35]. Conversely, it has been shown that the MR effect of the plate-like magnetic particles used in MR grease is significantly lower compared to the spherical magnetic particles-based MR grease. This is due to the lower magnetic saturation occurring from the plate-like magnetic particles, which limits the MR effect. Furthermore, it has been found that magnetic particles with anisotropic properties can show faster transient responses compared to the spherical- particles [36]. The importance of the transient response properties in the MR materials lies in the evolution of the chain structure formation of the magnetic particles in different media under the influence of a magnetic field, which will directly affect the responsiveness and performance of the MR devices and systems [14,37]. On top of that, this property is vital for the design optimization and precise control of MR applications [38]. 

Comprehensive investigation of the field-dependent rheological properties of bidisperse MRGs is considerably rare. Moreover, comparative examination of the apparent viscosity, storage modulus, and transient response between bidisperse MRGs with different weight percentage and MRG with spherical particles has not been reported so far. Many studies have considered the transient response of MR fluids in MR devices such as actuators, dampers, and brakes [39,40,41]. Two of the most significant material properties of MRG are fast transient response and high MR effect, which are directly related to the control performance of MRG-based devices and systems. Consequently, the technical contribution of this work is to comprehensively investigate the field-dependent behaviors of bidisperse MRGs which are composed of a combination of two different particle shapes and to compare those with mono-shaped MRG. Firstly, five different MRG samples with different weight percentages of spherical particles and plate-like particles are compared. Then, after explaining the testing methods of the MRG samples, the field-dependent apparent viscosities of MRG samples are measured under different strain amplitude and excitation frequency. Subsequently, the field-dependent storage and loss moduli of MRG samples are investigated in terms of their MR effect, which can be evaluated from the initial and maximum values. The transient response of mono-shaped and bidisperse MRG samples are investigated under a stepwise magnetic field using an oscillatory shear rheometer. In each property test, a comparative assessment of the five MRG samples is made in detail.

## 2. Results and Discussions

### 2.1. Apparent Viscosity

**Figure 1:**
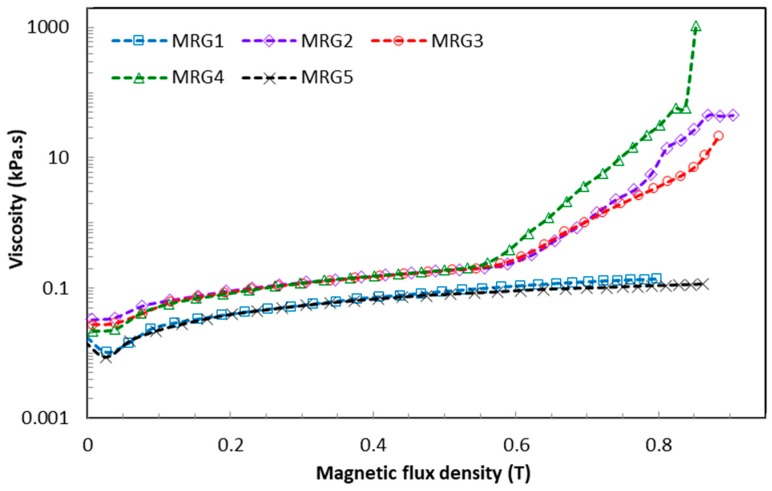
Apparent viscosity of magneto-rheological greases (MRGs) as a function of magnetic flux density.

Figure 1 shows the effect of the two particle shapes on the apparent viscosity of MRG samples as a function of magnetic flux density. As expected, the apparent viscosity of MRG samples is increased with increasing magnetic field. It has been found that MRG consisting of the mono-shaped CI particles has lower apparent viscosity compared to the bidisperse MRGs. It is also observed that MRG5 has the lowest apparent viscosity (0.1139 kPa.s), followed by MRG1 (0.1357 kPa.s), MRG3 (21.43 kPa.s), MRG2 (44.58 kPa.s), and MRG4 (1051 kPa.s). Theoretically, bidisperse MRGs will form strong and thick chain structures due to the anisotropy properties of the plate-like CI particles. The close packing between the plate-like and spherical CI particles hinders the particles from freely oriented in the medium without external force. Therefore, higher force is required for the particles to flow against the medium to form chain structures with the presence of shear rate and magnetic field. This can be seen clearly through the reduction of the void between CI particles [42]. A significant void reduction in the plate-like CI particles is expected compared to the spherical CI particles, owing to the larger diameter of anisotropy shape. As a result, the higher apparent viscosity is observed in the bidisperse MRG samples compared to the mono-shaped MR grease. The most concerning issue regarding the MRG is the initial apparent viscosity, which is typically higher compared to conventional MR fluids [21,32]. It is identified that the apparent viscosity of MRG, which is mostly dependent on the CI particles, can be altered by changing the size, shape, and fractions of the particles suspended in the medium. In addition, effective factors such as shear rate and particle–medium and particle–particle interactions should be taken into consideration. It can be seen from Figure 1 that the initial apparent viscosity of MRG5 (0.0148 kPa.s) is lower than MRG1 (0.0177 kPa.s) for the mono-shaped MRG. It is remarked here that unlike MR fluid, MRG is a non-Newtonian suspension that has yield stress in the absence of a magnetic field owing to the thixotropy properties of the grease which can retard the free movement of the CI particles. At the beginning of the applied magnetic field, the collision and cohesion forces between plate-like CI particles is smaller than for the spherical CI particles. In short, the particles–medium interaction, which is called the viscous force, is stronger than the particle–particle interactions—dipole interaction—at low magnetic field strength [43]. Moreover, the bedding position of the plate-like particles on the medium under the influence of gravity also slows down the formation of particle chain structures with the presence of shearing factor [44]. Thus, when a certain amount of magnetic force is exceeded, the yield stress and viscous force are required from the plate-like CI particles and they tilt and align to form strong and stable chain structures along the direction of the magnetic field [43,45,46,47].

**Figure 2:**
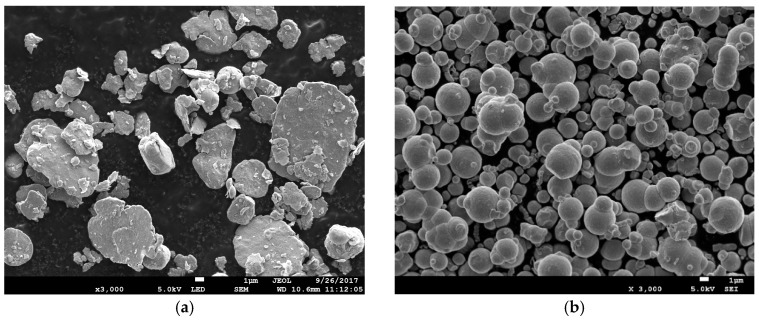
Scanning electron microscopy (SEM) morphology of different shapes of carbonyl iron (CI) particles; (**a**) plate-like and (**b**) spherical.

**Figure 3:**
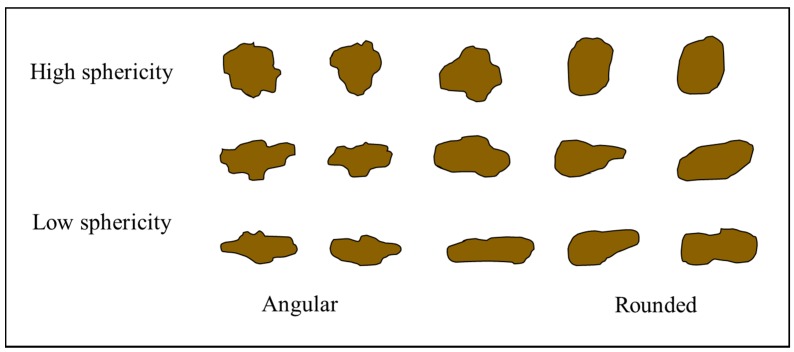
Alignment of particle chains corresponding to the applied magnetic field.

The different shapes and sizes of both CI particles have been observed under field emission scanning electron microscope (FESEM) as depicted in Figure 2. Thus, the rotation and alignment of the differently shaped CI particles subjected to the magnetic field in grease medium can be illustrated as shown in Figure 3. This phenomenon significantly reduces the initial apparent viscosity of the plate-like CI particles. The anisotropic CI particles prove that the reduction in the initial apparent viscosity of MRG can be achieved through utilizing different shapes of CI particles. Furthermore, it has been observed that the apparent viscosity of the bidisperse MRGs can be abruptly increased with an applied magnetic field of 0.5 T. This phenomenon is different in MRG comprised of mono-shaped CI particles. In addition, it has also been observed that MRG1 has reached its highest magnetic flux density at 0.8 T, which is the lowest compared to MRG2, MRG3, MRG4, and MRG5, in which the highest magnetic flux goes up to 0.9 T. This is because of the apparent viscosity parameter is significantly responsive to the anisotropy particle shape, about 20% more so than for the spherical CI particles [48]. It is acknowledged that the particles with anisotropic properties which can produce one or more axes in the presence of magnetic field tend to align in the long configuration following to the direction of magnetic field. Therefore, strong and hard chain alignments are created in the bidisperse MRGs. Additionally, the plate-like CI particles have a smaller demagnetized factor due to the spatial inhomogeneity of magnetization in their long direction [49]. In contrast, spherical CI particles with isotropic properties have no preferential direction for their magnetic moment unless there is an applied magnetic field, which normally aligns in one direction. The alignment of chain structure formed by spherical CI particles can be more easily disrupted and distorted compared to the plate-like CI particles. Consequently, this phenomenon directly contributes to the enhancement of the mechanical properties of the bidisperse MRGs. 

### 2.2. Storage and Loss Moduli

**Figure 4:**
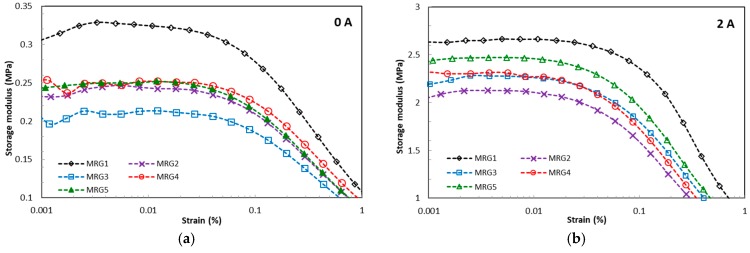
Storage modulus of MRG as a function of amplitude strain; (**a**) off-state, (**b**) on-state condition.

The field-dependent viscoelastic properties of MRGs are represented by the elasticity, energy dissipation, and damping factor under the deformation of chain structures due to the amplitude strain and frequency excitation. Recently, the contributions to understanding of particle interaction to the changes of viscoelastic behaviour have been taken into consideration. These properties have been acknowledged by researchers as vital parameters to evaluate both minimum and maximum requirements of physical forces, such as torque, in many dynamic systems. Figure 4 presents the effect of amplitude strain towards the storage modulus for MRG samples under different magnetic fields. From observation, MRG1 has the highest linear viscoelastic (LVE) region compared to the other MRG samples. The LVE region for MRG1 is improved from 0.03% to 0.1% by increasing the strength of the magnetic field. In contrast, there is no significant change in the LVE region between mono-shaped MRG5 and bidisperse MRG with γ = 0.04% under the influence of magnetic fields. More than that, the LVE region for bidisperse MRG is complicated to determine beyond 3 A. It is noted that the particles shape can be deduced as a factor that contributes to the changes of the LVE region. This happened due to the distinctive chain structures’ strengths between the different shapes of CI particles. It is known that the formation of particle chain structures is dynamically and continuously formed and distorted with the presence of amplitude strain. Moreover, stable chain structures are also affected by the lubrication factor of grease when amplitude strain is applied. This condition reduced the compatibility between the CI particles and the medium, as well as the average length of the chain structures which indirectly limited the LVE region of MRG. Figure 4 shows that the MRG comprised of plate-like CI particles (MRG2, MRG3, MRG4, and MRG5) has lower storage modulus compared to MRG1. Theoretically, CI particles with anisotropic shape have higher storage modulus compared to spherical-shaped particles credited to their anisotropic axes and larger surface contact area. However, due to the gravity factor, the plate-like CI particles favourably align in the bedding position and do not orientate according to their elongated axis in the medium [44,46]. Furthermore, the restriction due to grease structures and the presence of the strain amplitude has increased the void between differently shaped CI particles. This position has limited the particle–particle and particle–medium surface contact area, and hence higher strain rates are required for the tilting of the plate-like CI particles compared to spherical CI particles. Consequently, the reduction in dipole–dipole interactions between differently shaped CI particles can be examined via the lower stiffness of the MRG samples and the Payne effect by increasing both the amplitude strain and magnetic field. Table 1 provides the viscoelastic performances of the MRG samples in the LVE region. 

**Figure 5:**
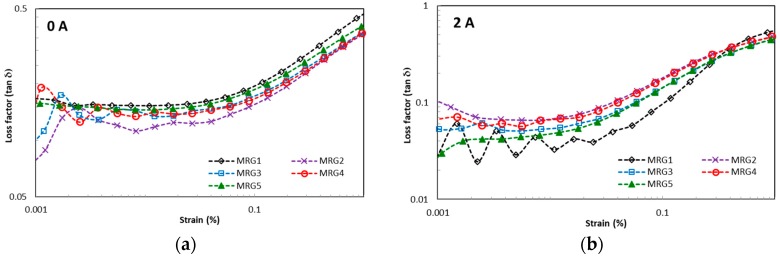
Loss factor of MRG samples as a function of amplitude strain; (**a**) off-state, (**b**) on-state condition.

Figure 5 shows the loss factor of MRG samples over amplitude strain, which represents the damping property of the samples. In the absence of a magnetic field, the bidisperse MR grease has a fluctuating loss factor compared to the mono-shaped MRG. In contrast, the fluctuation of the loss factor is more noticeable in mono-shaped MRG in the on-state condition; specifically, MRG1 with the presence of a magnetic field at amplitude strain <0.1%. Damping property is a key parameter to evaluate whether the dissipation of bidisperse MRG is higher than mono-shaped MRG in the presence of a magnetic field. This behaviour occurs due to the larger surface contact area of the plate-like CI particles. The combination of both CI particle shapes in MRG may hinder particle rotation in the medium [50]. This result reveals that the Payne effect is pronounced in MRG comprised of spherical-shaped CI particles compared to the anisotropic CI particles with increasing amplitude strain and magnetic field. 

**Figure 6:**
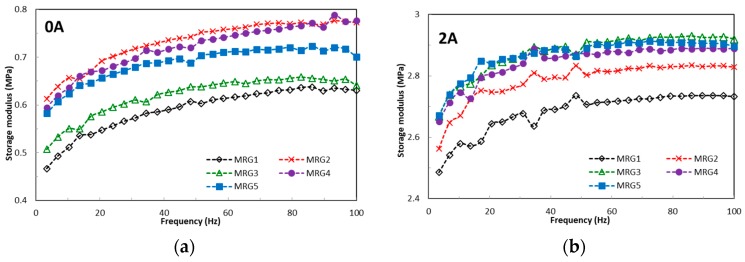
Storage modulus of MRG as a function of frequency (**a**) off-state, (**b**) on-state condition.

**Figure 7:**
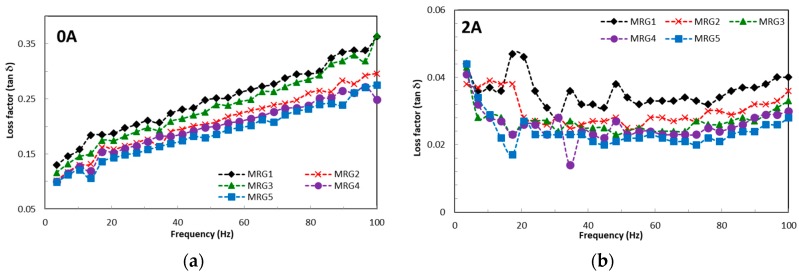
Damping factor of MRG as a function of frequency; (**a**) off-state, (**b**) on-state condition.

The effect of frequency on the storage modulus and damping factor are displayed in Figure 6 and Figure 7. The lowest storage modulus is acquired from MRG1 in both the absence and presence of a magnetic field. It has also been observed that the storage moduli of all MRG samples increase as the magnetic field increases. This directly indicates that the particles’ shape is pronounced under the influence of frequency. Moreover, the increment of the storage modulus, which represents better elasticity, is achieved with anisotropic particles in which the chain alignment is well determined. It has been found from Figure 6 that the loss factor all of MRG samples increased with the increasing of frequency in the absence of a magnetic field. In contrast, the loss factor of MRG samples is considered stagnant with increasing frequency when subjected to the magnetic field. Furthermore, anisotropic particles show a better damping property than spherical CI particles. In this study, the spherical CI particles produced larger energy dissipation due to the improvement in the friction between particles and the grease medium. Moreover, interfacial slipping also contributes to the larger energy dissipation in spherical shapes [51]. Meanwhile, the plate-like CI particles favour aligning in the bedding position, which can lead to the reduction of friction between anisotropic particles. This phenomenon proves that the anisotropic particles have better absorbing properties which are appropriate for vibrational applications. 

### 2.3. Transient Response

**Figure 8:**
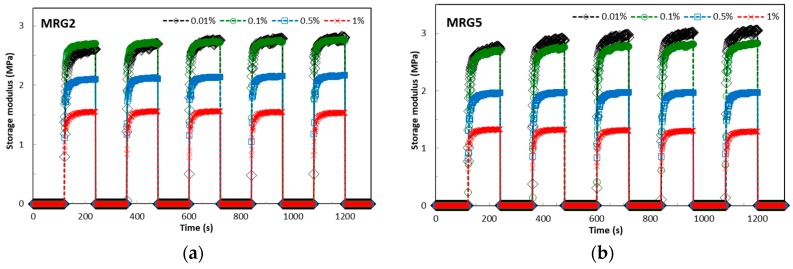
Transient responses of MRG samples with influence of variation actuating amplitude strains.

The transient response of mono-shaped and bidisperse MRG samples was investigated under a step-wise magnetic field in order to gain better insight on the chain-like formation of the different shapes of CI particles in the grease medium. Figure 8 shows the transient response of the MR grease samples under transient response with variation of amplitude strain towards the storage modulus. The main purpose of this experiment was to ensure that the chain microstructure of the MRG is not destroyed through actuating amplitude strain as the oscillatory shear test is implemented during long operation. It is observed that the mono-shaped MRG shows higher storage modulus than bidisperse MRG at various actuating amplitude strains under the influence of transient response. In addition, the transient responses of the MRG samples are calculated based on 63.2% output of the steady state value [52]. The mono-shaped MRG with plate-like CI particles exhibits the longest transient response of six seconds at an actuating amplitude strain of 0.01%. Subsequently, the transient response is improved to two seconds after applying an actuating amplitude strain above 0.5%. Meanwhile, mono-shaped spherical CI particles and bidisperse MRG show transient responses of five seconds and improved to two seconds for 0.01% and above 0.1% actuating amplitude strain, respectively. In general, the transient responses of the MRG samples decreased with increasing actuating amplitude strain. The mono-shaped MRG with spherical CI particles (MRG1) has the highest storage modulus at various actuating amplitude strains. However, mono-shaped MRG with plate-like CI particles (MRG5) shows the highest at 0.01% amplitude strain and declined abruptly with increasing actuating amplitude strain above 0.5%. In addition, the trend of storage modulus for MRG1, MRG2, MRG3, and MRG4 was a slight increase from 0.01% to 0.1% actuating amplitude strain. It then declined with further increasing strain. Conversely, MRG5’s storage modulus is reduced by increasing the actuating amplitude strain. This phenomenon can be closely related to the uniformity of CI particles dispersed in the grease. In this case, there are many short chain structures during the stepwise increment [53]. Moreover, it is noted that the anisotropic shape of CI particles results in a better time response that the isotropic spherical shape [36]. However, the restrained motion from the grease structures and orientation of the plate-like CI particles may hinder the chain structuring process. This condition leads to a reduction in storage modulus under periodical stepwise magnetic fields. 

As mentioned above, the mono-shaped MRG with plate-like CI particles has the least friction and slippage due to their bedding plane orientation. In addition, the strength of the chain formation established for MRG5 is strong compared to MRG1. Henceforth, with the increasing of actuating amplitude strain in the presence of a magnetic field, the chain structures are easily disrupted, directly reducing the storage modulus. Furthermore, it is observed that the bidisperse MRG has an enhanced LVE region of the mono-shaped MRG with the plate-like CI particles, which in turn indirectly increases the storage modulus. Based on authors’ previous studies, the average diameter for the plate-like and spherical CI particles are 6.2 µm and 5 µm, respectively [33]. Theoretically, the small-diameter spherical CI particles are inclined to fill the void between the larger plate-like CI particles in the grease medium [42]. In this study, it is believed that the small diameter of spherical CI particles is adsorbed on the rough surface of larger plate-like CI particles, which are favour orientation in bedding plane. This condition indirectly reduces the friction and slippage of spherical CI particles towards the rough surfaces of plate-like CI particles. As a result, the chain formation in bidisperse MRG becomes stronger and hence the field-dependent properties, such as storage modulus and LVE region over transient response, can be enhanced.

## 3. Materials and Methods

In this study, two shapes of magnetic particles—spherical and plate-like CI (carbonyl iron) particles—were used to prepare the bidisperse MR grease samples. The spherical CI particles with OM grades were purchased from BASF, Germany with the average diameter in the range of 3.9 to 5.2 µm and density of 7.874 g cm^−3^. The plate-like CI particles were prepared using a high-energy rotary ball mill machine used in previous works [8,33]. The spherical CI particles were milled with the presence of a zirconia ball as the grinding medium at a ball-to-powder ratio of 20:1. The speed of the rotary ball mill was fixed at 180 rpm for 40 hours. Herein, commercial lithium grease (NPC Highrex HD-3 Grease) provided by Nippon Koyu Ltd, Japan was chosen as the suspension medium to produce bidisperse MRG samples. The density and viscosity of the grease specified by the manufacturer is 0.92 g/cm^3^ and 190 cSt, respectively. A series of bidisperse MR greases were prepared by dispersing spherical and plate-like CI particles in the grease medium via a mechanical stirrer. The homogeneity of the samples was improved by stirring for 2 hours. In this study, a constant total weight fraction of CI particles was chosen as 70 wt% to prepare the samples. The details of sample composition are listed in Table 2.

In this study, the viscoelastic properties of the samples were characterized by a commercial parallel-plate rheometer (MCR 302, Anton Paar) equipped with magnetic field support by an MR device (MRD70/1T). For this purpose, one mL of the samples was used to fill up the base plate and the oscillatory mode was performed using a measuring plate with a diameter of 20 mm and a constant gap distance of 1 mm. The samples were characterized at on-state condition by varying the magnetic flux density from 0 to 0.8 T and adjusting the coil-applied current in the range of 0 to 5 A. The viscoelastic properties of elasticity, energy dissipation, and damping factor were analyzed through oscillation test under 0.01% strain amplitude and excitation frequency of 1 Hz. Furthermore, the transient response under a step-wise magnetic field was performed at first for 120 s for the off-state condition. A magnetic field of 0.7 T was instantaneously applied to the samples for the interval of 120–240 s for the on-state condition and instantaneously removed at 240 s. The step-wise interval was repeated for five loops. The dimensionless storage moduli of the samples were then calculated and analyzed. It is noted that all tests were undertaken at room temperature in this work.

## 4. Conclusions

In this work, the field-dependent rheological properties and transient responses of three bidisperse MRGs and two types of mono-shaped MRGs have been experimentally investigated and evaluated. Five different MRG samples were prepared by adjusting the percentages of two different particle shapes; spherical and plate-like CI particles. From the tests, the following results have been obtained.

(1) The apparent viscosity results showed that the presence of anisotropic-shaped CI particles was able to reduce the initial apparent viscosity; 0.1139 kPa.s for MRG5, due to its orientation in the bedding plane. Conversely, the bidisperse MRG showed enhancement in the initial apparent viscosity as well as stiffness compared to the mono-shaped MRG, with 21.43 kPa.s, 44.48 kPa.s, and 1051 kPa.s for MRG3, MRG2, and MRG4 respectively. This indicates that the bidisperse MRG has an improved particle structuring process by strengthening the chain structures and reducing the void in the thixotropic medium.
(2) The LVE region for the mono-shaped MRG comprised of spherical CI particles improved in the presence of a magnetic field from 0.03% to 0.1%. Meanwhile, the mono-shaped plate-like and bidisperse MRGs have shown limited LVE region with 0.04% magnetic field. This LVE region indirectly limits the performance of the MRG in devices. From this study, MRG5 exhibited the highest MR effect of about 250%, followed by the mono-shaped spherical CI particles (240%) and bidisperse MRG (220%). The MR effect of the bidisperse MRG is lower than expected due to the bedding orientation of the plate-like CI particles in the medium and restriction of the grease structure.
(3) The transient response was evaluated in this study, which is vital to device performances. The results showed that MRG1, MRG2, MRG3, and MRG4 have better storage modulus at LVE region of 0.1% with transient responses of two seconds during stepwise magnetic field application compared to MRG5, which at 0.01% had a response time of six seconds.

The results presented in this work show that the field-dependent rheological properties and transient response of MRG can be altered by adjusting the weight percentage of differently shaped CI particles. Therefore, the presented results will be very helpful in deciding which MRG to use for a certain application. One can choose an MRG which has a low storage modulus in the absence of the magnetic field, but very high storage modulus with fast onset when a magnetic field is applied. Based on the presented results, in general, the shape of soft magnetic particles inside MR materials strongly affects the responsiveness of the materials to magnetic fields. This fact will be useful for the design of MR-based devices.

## Figures and Tables

**Table 1:** Properties of MRG samples.

Samples	Initial Storage Modulus, G’_0_ (MPa)	Absolute MR Effect (MPa)	Relative MR Effect (%)
MRG1	0.76383	1.83474	240.20
MRG2	0.72047	1.71398	237.90
MRG3	0.77925	1.72605	221.50
MRG4	0.75217	1.70625	226.84
MRG5	0.73861	1.89573	256.66

**Table 2:** Composition of the bidisperse MRGs (wt%).

Samples	Spherical CI Particles	Plate-Like CI Particles	Grease
MRG1	70	0	30
MRG2	50	20	30
MRG3	35	35	30
MRG4	20	50	30
MRG5	0	70	30

## Data Availability

Data will be made available on request.
